# Identification of differentially expressed ER stress-related genes and their association with pulmonary arterial hypertension

**DOI:** 10.1186/s12931-024-02849-4

**Published:** 2024-05-24

**Authors:** Qi Yang, Banghui Lai, Hao Xie, Mingbin Deng, Jun Li, Yan Yang, Juyi Wan, Bin Liao, Feng Liu

**Affiliations:** 1 Department of Cardiovascular Surgery, The Affiliated Hospital, Metabolic Vascular Diseases Key Laboratory of Sichuan Province, Key Laboratory of Cardiovascular Remodeling and Dysfunction, Southwest Medical University, Luzhou, Sichuan 646000 P.R. China; 2 Laboratory of Medical Electrophysiology, Ministry of Education & Medical Electrophysiological Key Laboratory of Sichuan Province, (Collaborative Innovation Center for Prevention of Cardiovascular Diseases), Institute of Cardiovascular Research, Southwest Medical University, Luzhou, Sichuan 646000 P.R. China

**Keywords:** Pulmonary arterial hypertension, Hub genes, Endoplasmic reticulum stress, Immune infiltration, Bioinformatic analysis

## Abstract

**Background:**

Pulmonary arterial hypertension (PAH) is a complex and progressive illness that has a multifaceted origin, significant fatality rates, and profound effects on health. The pathogenesis of PAH is poorly defined due to the insufficient understanding of the combined impact of endoplasmic reticulum (ER) stress and immune infiltration, both of which play vital roles in PAH development. This study aims to identify potential ER stress-related biomarkers in PAH and investigate their involvement in immune infiltration.

**Methods:**

The GEO database was used to download gene expression profiles. Genes associated with ER stress were obtained from the MSigDB database. Weighted gene co-expression network analysis (WGCNA), GO, KEGG, and protein-protein interaction (PPI) were utilized to conduct screening of hub genes and explore potential molecular mechanisms. Furthermore, the investigation also delved into the presence of immune cells in PAH tissues and the correlation between hub genes and the immune system. Finally, we validated the diagnostic value and expression levels of the hub genes in PAH using subject-workup characterization curves and real-time quantitative PCR.

**Results:**

In the PAH and control groups, a total of 31 genes related to ER stress were found to be differentially expressed. The enrichment analysis revealed that these genes were primarily enriched in reacting to stress in the endoplasmic reticulum, dealing with unfolded proteins, transporting proteins, and processing proteins within the endoplasmic reticulum. EIF2S1, NPLOC4, SEC61B, SYVN1, and DERL1 were identified as the top 5 hub genes in the PPI network. Immune infiltration analysis revealed that these hub genes were closely related to immune cells. The receiver operating characteristic (ROC) curves revealed that the hub genes exhibited excellent diagnostic efficacy for PAH. The levels of SEC61B, NPLOC4, and EIF2S1 expression were in agreement with the findings of bioinformatics analysis in the PAH group.

**Conclusions:**

Potential biomarkers that could be utilized are SEC61B, NPLOC4, and EIF2S1, as identified in this study. The infiltration of immune cells was crucial to the development and advancement of PAH. This study provided new potential therapeutic targets for PAH.

**Supplementary Information:**

The online version contains supplementary material available at 10.1186/s12931-024-02849-4.

## Introduction

PAH, also known as pulmonary arterial hypertension, is a complex and life-threatening condition that involves various factors leading to increased pressure in the arteries of the lungs, ultimately affecting the right ventricle [1]. The development of PAH involves a combination of genetic predispositions and environmental influences. Central to the pathogenesis of PAH is the interaction between endoplasmic reticulum (ER) stress and systemic inflammation. ER stress, often triggered by protein misfolding and accumulation within the ER, activates the unfolded protein response (UPR). This response, while initially protective, can become maladaptive, releasing inflammatory cytokines that contribute to vascular injury and exacerbating ER stress by overwhelming the ER’s folding capacity, thus perpetuating a cycle of damage. This feedback loop is crucial in driving the pathophysiological changes observed in PAH, including the development of neointimal lesions and plexiform lesions, hallmark features of advanced PAH [2–4]. Numerous proposed potential mechanisms involve the malfunction of endothelial cells in the pulmonary blood vessels, genetic mutations, inflammation, immune reactions, remodeling of blood vessels, blood clot formation, abnormalities in ion channels, and additional factors [3–7]. Basic therapy and targeted drug therapy are the main approaches used to treat pulmonary arterial hypertension (PAH) at present. Although these treatments can effectively relieve patients’ clinical symptoms, they do not have the ability to halt the progression of the disease or substantially decrease mortality rates [8, 9]. Further research on mechanisms and biomarkers is needed to enable early diagnosis and more effective treatments.

The response to endoplasmic reticulum (ER) stress is characterized by the initiation of signaling pathways, including the unfolded protein response (UPR), the ER overload response, and the caspase-12 mediated apoptosis pathway in response to disrupted protein folding, accumulation of misfolded proteins, calcium ion imbalance, and calcium levels within the ER. This response can result in cytoprotective outcomes, cellular damage, or apoptosis [10, 11]. According to recent research, ER stress is also extensively implicated in cancer, cardiovascular disorders, diabetes, and various other ailments [12–14]. ER stress is a prevalent factor in PAH, and there is evidence indicating its involvement in the proliferation of smooth muscle cells in the pulmonary artery (PASMCs) and the enhancement of the inflammatory response. The abnormal growth and resistance to cell death of PASMCs play a crucial role in the pathological changes to blood vessels seen in PAH [15]. Therapeutically, targeting ER stress represents an innovative approach for the clinical treatment of PAH. Research shows that 4-phenylbutyrate and similar chemical chaperones can alleviate ER stress and potentially treat PAH in animal models. This is achieved by inhibiting the proliferation of PASMC, promoting apoptosis, and decreasing the expression of factors related to ER stress [16]. However, clinical testing remains absent. However, these preliminary results suggest that decreasing ER stress may offer a hopeful approach for treating PAH.

High levels of various cytokines, chemokines, and autoantibodies have been confirmed in animal models of PAH and in PAH patients. Significantly, the degree of perivascular inflammation in the lungs in PAH is associated with pulmonary hemodynamics, remodeling of blood vessels, and clinical results [17]. A recent study using impartial computational flow cytometry analysis of the lungs in individuals with idiopathic PAH and healthy donors revealed that macrophage recruitment can be enhanced [18]. Increased levels of C-reactive protein enhance the proliferation of smooth muscle cells in the pulmonary arteries, trigger the secretion of endothelin, decrease the production of nitric oxide, and worsen vascular remodeling. Moreover, C-reactive protein further stimulates inflammatory responses, resulting in the secretion of additional inflammatory agents and worsening the state of individuals with PAH [19]. Although the role of ER stress and immune responses in the development of PAH is well-known, there is still a lack of research on their synergistic impact, which requires further investigation.

For this investigation, we utilized bioinformatics methods to examine chip data sourced from the GEO database. Our aim was to pinpoint hub genes that are differentially expressed and associated with ER stress in PAH. Additionally, we explored the functions of endoplasmic reticulum stress and the infiltration of the immune system in PAH. The results of our study indicate possible indicators and treatment targets for PAH.

## Materials and methods

### Data acquisition

The dataset GSE113439 based on the GPL6244 platform was downloaded from the National Center for Biotechnology Information database (https://www.ncbi.nlm.nih.gov/) for further analysis. The dataset included lung tissue transcriptome data from 15 PAH patients and 11 normal control groups.

### Detection of genes with differential expression

The Limma software package (version 3.58.1) was employed for identifying differentially expressed genes (DEGs) between the PAH group and the control group. DEGs with an absolute log fold change greater than 0.585 and a *p*-value less than 0.05 were deemed statistically significant. “ggplot2” and “pheatmap” packages were used to plot volcanoes and heat maps of DEGs.

### Weighted gene co-expression network analysis (WGCNA)

The gene co-expression networks were constructed using the R package ‘WGCNA’ by utilizing the merged database. Initially, the software R was employed to calculate a gentle threshold power β, which was then applied to elevate the co-expression similarity for determining the degree of neighborliness. Next, the mean linked hierarchical clustering technique was employed to group genes exhibiting comparable patterns into indistinguishable modules. For the merging of modules, we employed a threshold of 0.25, ensuring that each module comprised a minimum of 30 genes. Modules associated with clinical shape were identified by utilizing correlations between branches of the clustering tree and various color phenotypes. The clinical traits of the modules were calculated using gene significance (GS) and module membership (MM). In conclusion, an examination was conducted on core modules that exhibited a strong correlation with clinical characteristics.

### Identification of ER stress-related DEGs (ER- DEGs)

The genes associated with ER stress (GOBP response to endoplasmic reticulum stress and GOBP regulation of endoplasmic reticulum stress) were acquired from the Molecular Signature Database v7.0 (MSigDB). The VennDiagram software package was used to obtain ER-DEGs linked to PAH by intersecting all DEGs, genes related to ER, and core module genes. The clusterProfiler (version 4.10.0) was utilized to conduct GO and KEGG pathway enrichment analyses, with entries having a *P*-value less than 0.05 deemed as statistically significant.

### Identification and correlation analysis of hub genes

The overlapping genes were uploaded to the STRING database to conduct PPI analysis, where the minimum interaction score was set to 0.4. The visualization was performed using Cytoscape 3.9.1, and the identification of hub genes was carried out by applying the MCC algorithm through the CytoHubba plug-in. Next, the software package ggplot2 was utilized to create box plots illustrating the gene expression profiles of central genes. For further analysis, we utilized the pROC software package to conduct ROC curve analysis on hub genes, with an AUC > 0.8 being deemed as the optimal diagnostic value. The circle software was utilized to examine the association between hub genes and optimal diagnostic value.

### Analysis of immune infiltration

The presence and growth of immune cells play a crucial role in the onset and progression of PAH. Consequently, we investigated if these central genes were associated with the infiltration of diverse immune cells. We evaluated the correlation of 28 immune cell infiltrates in the GSE113439 sample by employing single-sample (ss) GSEA (version 1.42.1).

### Animal model

The Ethics Committee for Animal Experiments of Southwest Medical University granted approval for this study. A total of sixteen male SD rats, aged 4 weeks and obtained from Luzhou Yinhui Biotechnology Co., Ltd., were randomly assigned to two groups: a model group (referred to as the PAH group, consisting of 8 rats) and a control group (referred to as the CON group, also consisting of 8 rats). The PAH group established a rat PAH model through complete removal of the left lung, while the CON group remained unoperated. For a duration of 4 weeks, the experimental animals were housed in identical conditions within an SPF-grade environment at the Animal Center of Southwest Medical University. Following a period of 4 weeks, the rats were administered anesthesia and a catheter was inserted through the right internal jugular vein to access the right ventricle. The BL-420 S Biofunctional Experiment System (Chengdu, China, Taimeng) was utilized to measure the right ventricular systolic pressure (RVSP). Subsequently, the rats used in the experiment were put to death, and their lung and heart tissues were gathered. The heart was partitioned into the right ventricle (RV), left ventricle (LV), and interventricular septum (S). Subsequently, the index for right ventricular hypertrophy (RVHI) was computed using the formula RVHI = weight _RV_/weight _LV+S_. H.E. staining was performed on formaldehyde-fixed, paraffin-embedded partial lung tissues obtained from experimental rats. Frozen lung tissues were used for subsequent qRT-PCR analysis using real-time quantitative polymerase chain reaction.

### qRT-PCR

To further examine the expression of central genes in lung tissues of rats with PAH, qRT-PCR was employed. TRIZOL was utilized to extract total RNA from lung tissues, which was then reverse transcribed into cDNA using a reverse transcription kit from Roche. The completion of qRT-PCR involved the utilization of SYBR green from Roche. The supplementary material S1 displays the primers utilized for amplification. The expression of the target gene was demonstrated as 2-ΔΔCt relative to the GAPDH gene. The experimental results were presented as the average plus or minus the standard deviation, and a *p*-value less than 0.05 was deemed to be statistically significant.

### Statistical analysis

Statistical analyses were conducted using R software (version 4.3.1) and GraphPad Prism 10.0.0. qRT-PCR assays were performed with three replicates. The data was analyzed and converted into bar graphs using GraphPad Prism. The analysis of differences between two independent groups was conducted using the Student’s t-test. Statistical tests were conducted on both sides, and a *p* value < 0.05, after correction, was deemed to be statistically significant.

## Results

### Identification of differential expressed and modular genes

**Fig. 1:**
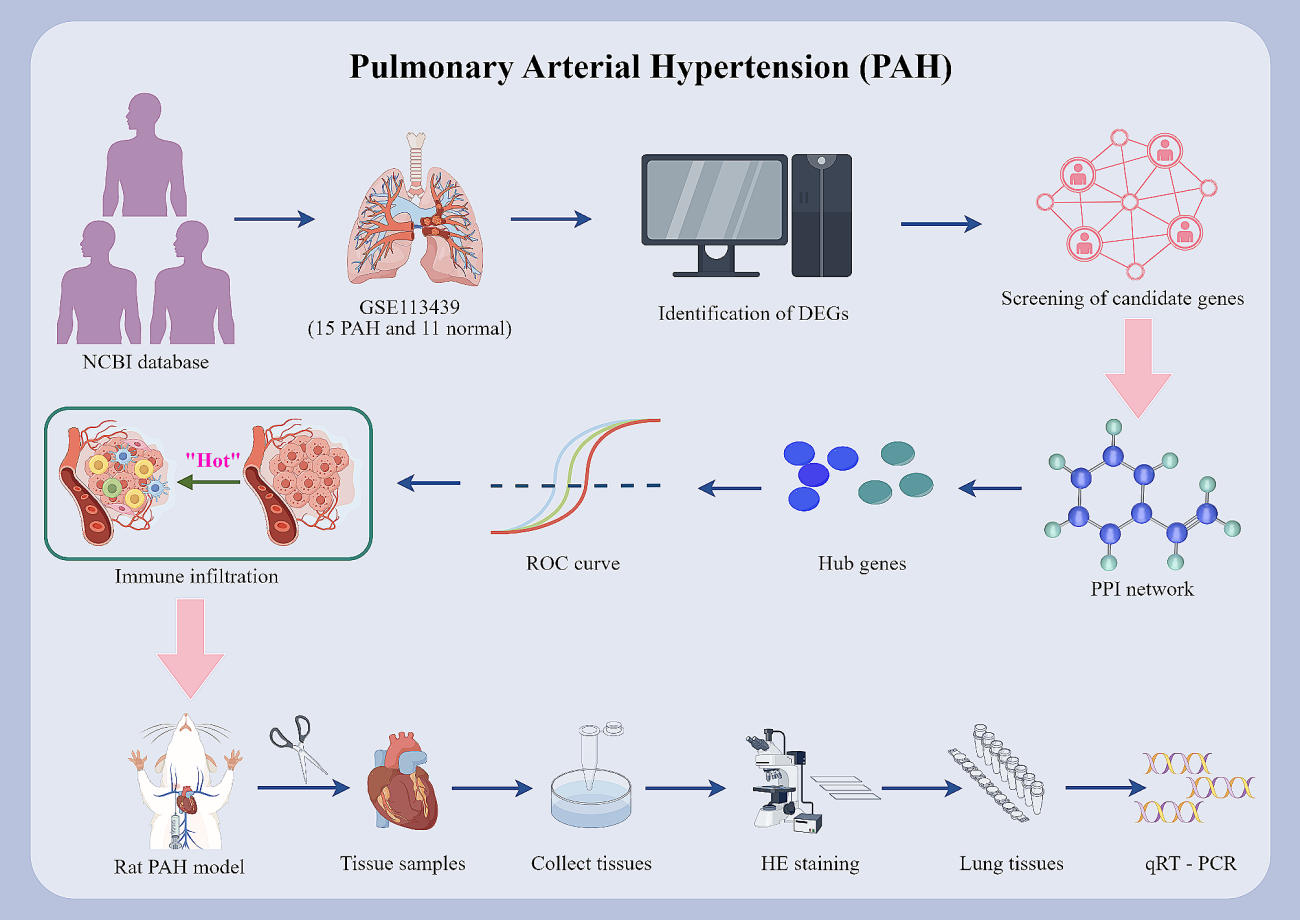
Schematic representation of the pathophysiological mechanisms involved in pulmonary arterial hypertension (PAH). This diagram illustrates the key processes contributing to the development and progression of PAH, including endothelial dysfunction, smooth muscle cell proliferation, and inflammatory responses. The figure highlights the interaction between endoplasmic reticulum (ER) stress and systemic inflammation, detailing how ER stress activates the unfolded protein response (UPR), which can exacerbate vascular injury and contribute to neointimal formation and plexiform lesions. Each pathway and its role in PAH pathogenesis are clearly labeled to facilitate understanding of the complex disease mechanisms

**Fig. 2:**
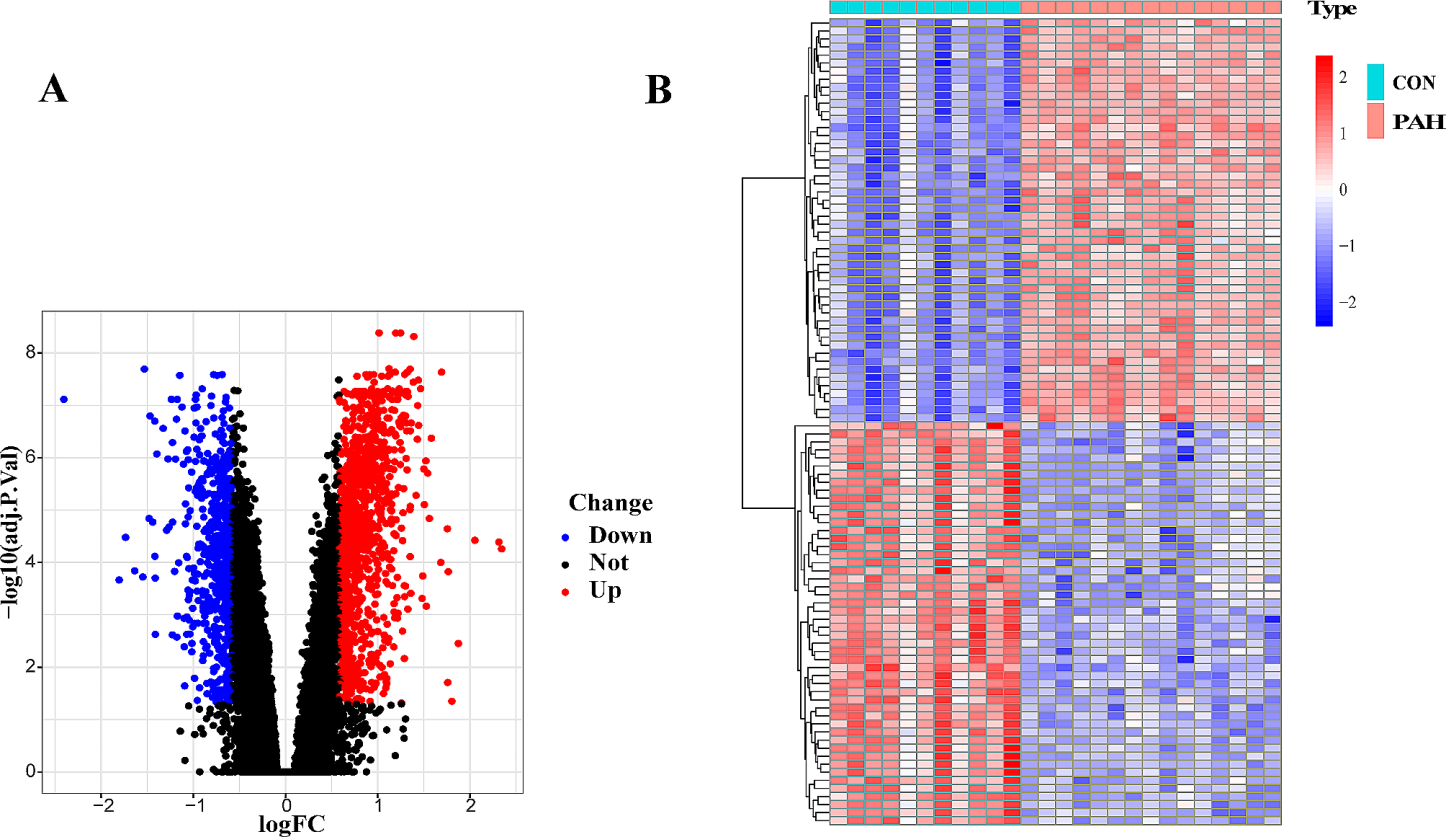
Differential genes expression in pulmonary arterial hypertension (PAH). **A**: Volcano plot illustrating differentially expressed genes (DEGs) between PAH and control groups. Upregulated genes are indicated by red dots, downregulated genes by blue dots, and genes with no significant expression change are represented by black dots. The x-axis displays the log2 fold change, while the y-axis represents the negative log10 of the adjusted *p*-value, underscoring both the magnitude and significance of expression changes. **B**: Heatmap showing the expression patterns of top DEGs across samples. Hierarchical clustering groups genes with similar expression profiles (y-axis) and segregates control (CON) from PAH samples (x-axis). The color gradient, from blue to red, indicates expression levels from downregulated to upregulated, with the color intensity reflecting the z-score normalized expression values

**Fig. 3:**
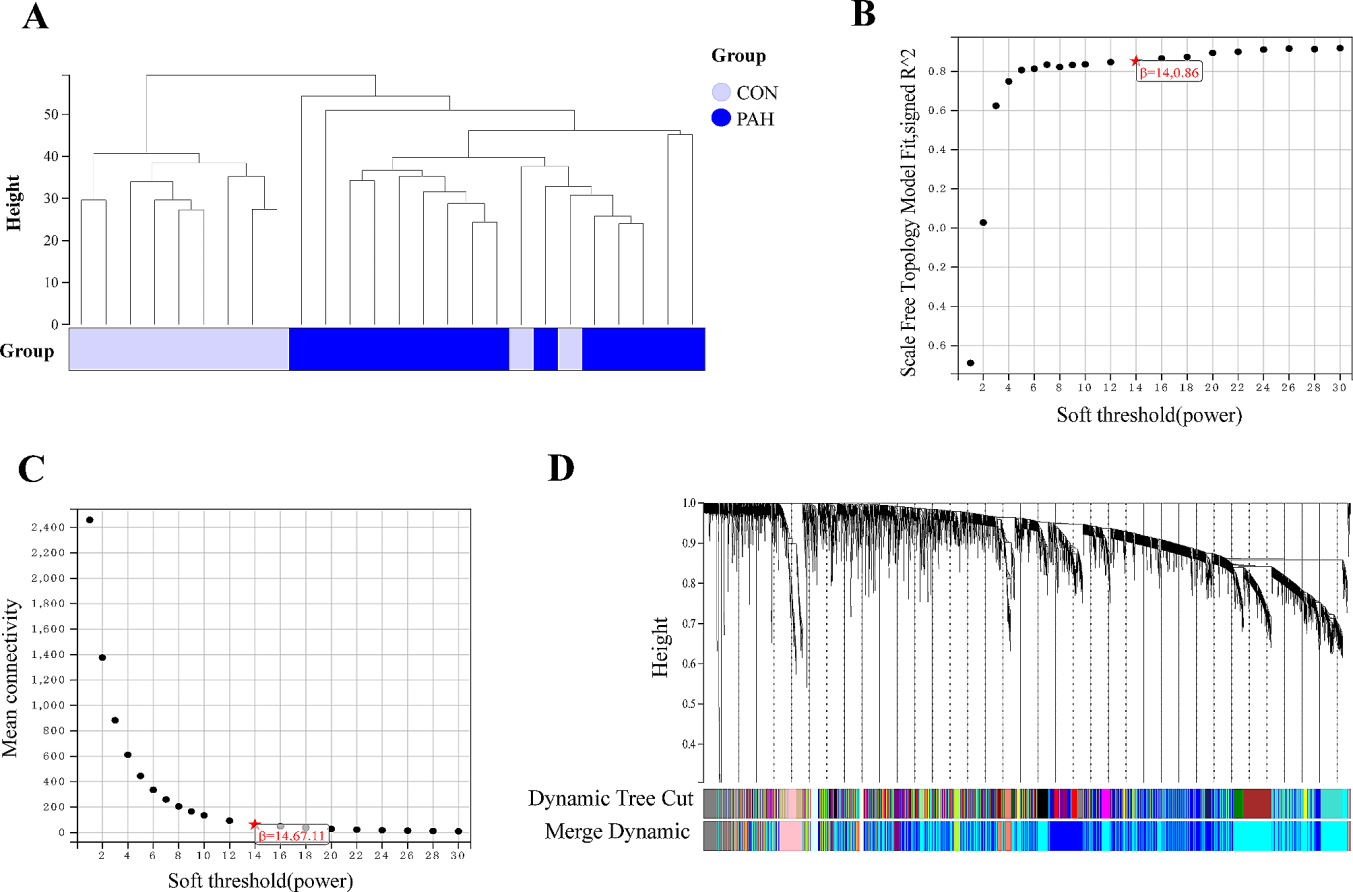
Weighted Gene Co-Expression Network Analysis (WGCNA) for the identification of gene modules associated with pulmonary arterial hypertension (PAH). **A**: Dendrogram produced by hierarchical clustering of samples based on gene expression profiles, showing the grouping of PAH (blue) and control (light purple) samples. **B**: Analysis of network topology for various soft-thresholding powers. The red line indicates the selected power (β) at which the scale-free fit index curve flattens out, suggesting a suitable model fit. **C**: Plot of mean connectivity as a function of the soft-thresholding power, with the red line marking the chosen power based on when the scale independence meets the criteria for a scale-free network. **D**: Dendrogram of all genes clustered based on a dissimilarity measure (1-TOM), with the resultant module colors displayed below. Each color represents a module of highly interconnected genes, with the dynamic tree cut illustrating the modules’ divisions

**Fig. 4:**
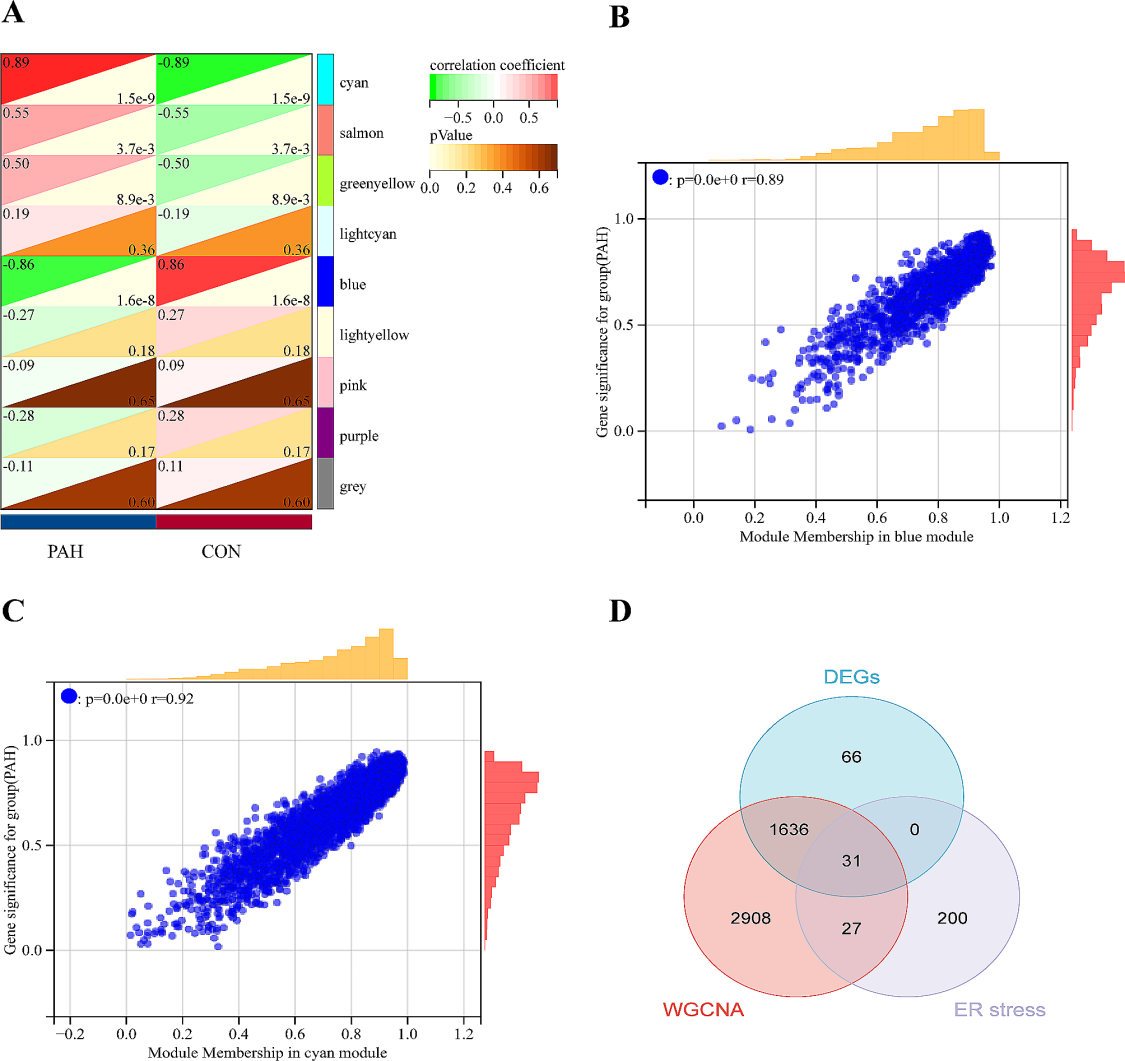
Module-trait relationships and identification of genes associated with pulmonary arterial hypertension (PAH). **A**: Heatmap depicting the correlation between gene modules and clinical traits (PAH and control). Each row corresponds to a gene module color-coded as per the legend, and each column represents a clinical trait. The color within the heatmap reflects the correlation coefficient, with the scale shown on the right, and the numbers in each cell represent the *p*-values, indicating the significance of the correlation. **B**: Scatterplot demonstrating the correlation between Module Membership (MM) in the blue module and Gene Significance (GS) for PAH. A high degree of correlation indicates that genes most central to the module are also highly significant for the trait. **C**: Scatterplot showing the correlation between MM in the cyan module and GS for PAH, with a similarly strong correlation suggesting that this module is significantly related to the disease. **D**: Venn diagram illustrating the overlap between differentially expressed genes (DEGs), genes identified through Weighted Gene Co-expression Network Analysis (WGCNA), and genes related to ER stress. The numbers in the intersections represent genes common to the groups, indicating potential key candidates involved in PAH pathogenesis

The overall process of the study is showed in Fig. 1. In the GSE113439 dataset, a total of 1733 differentially expressed genes (DEGs) were found between the PAH and control groups. Among these DEGs, 1127 genes were up-regulated while 606 genes were down-regulated, as shown in Fig. 2A and B). In the WGCNA analysis, the dendrogram encompassed the gene sets of all samples(Fig. 3A and D). The threshold for softness was established at 14 (R2 = 0.86), and the scale-free network was constructed (Fig. 3B and C), which identified nine modules (Fig. 4A). The blue and cyan modules were chosen for further investigation based on a correlation coefficient that exceeded 0.8 (Fig. 4B and C). Specifically, MM represents the inclusion of a gene within a module, quantified as the closeness of the gene to the module eigengene, which is the principal component of the module’s expression matrix. GS measures the correlation between gene expression and the clinical traits associated with PAH, providing a numerical indication of each gene’s relevance to the traits being studied.

### Screening and functional enrichment analysis of candidate genes

**Fig. 5:**
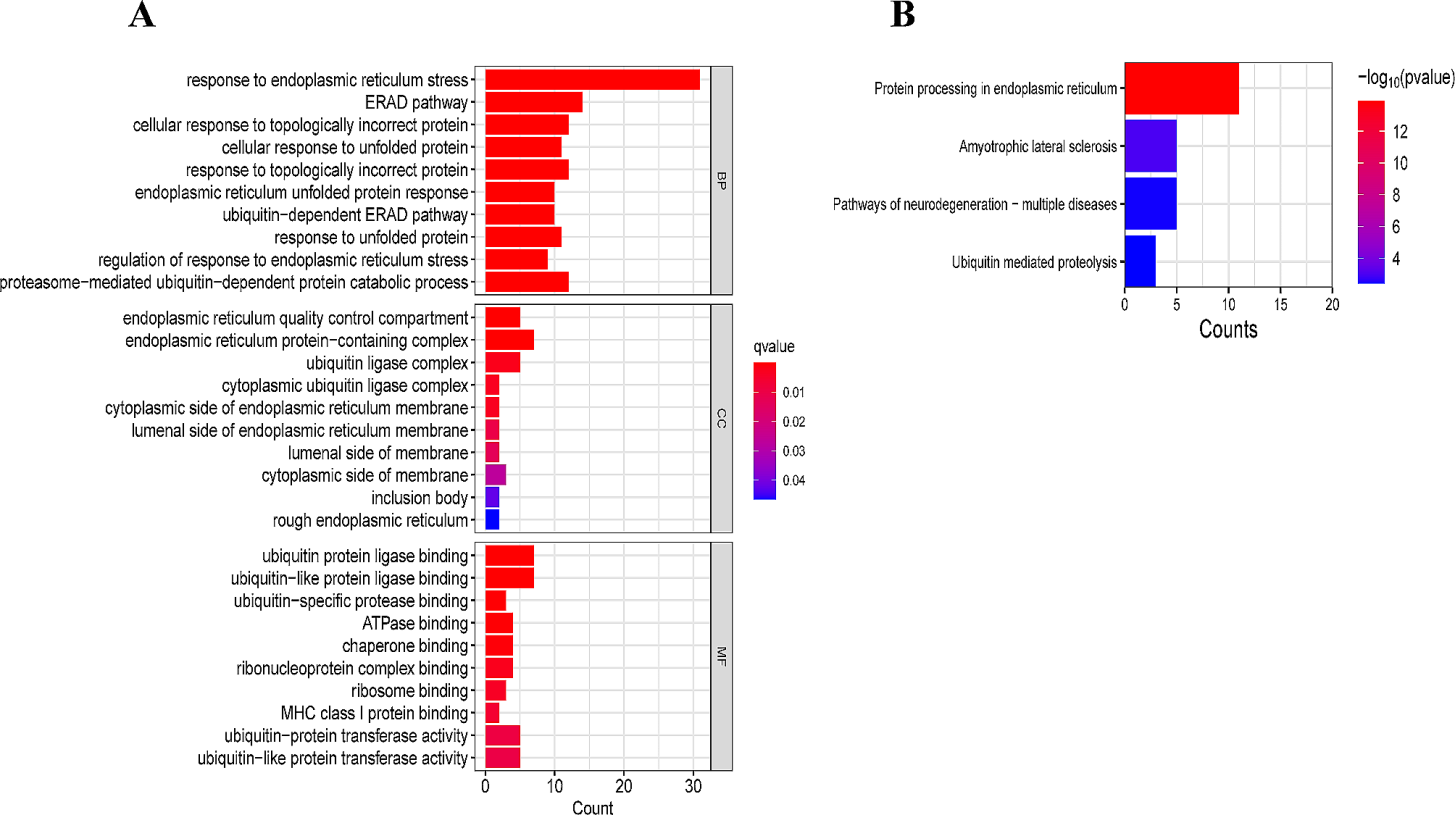
Gene Ontology (GO) and Kyoto Encyclopedia of Genes and Genomes (KEGG) pathway enrichment analyses of genes associated with pulmonary arterial hypertension (PAH). **A**: Bar chart of the GO term enrichment analysis results categorized into biological processes (BP), cellular components (CC), and molecular functions (MF). The length of each bar represents the gene count associated with each term, with color intensity indicating the q-value, a measure of significance after correcting for multiple testing. **B**: KEGG pathway enrichment analysis bar chart showing the pathways most significantly associated with the hub genes. The number of genes is denoted by the bar’s length, while the color gradient represents the -log10(*p*-value), highlighting the statistical significance of the enrichment

Figure 4D showed the identification of 31 candidate hub genes related to PAH and ER stress by intersecting 1733 DEGs, 4602 modular genes, and 258 ER stress-associated genes. Using GO and KEGG pathway analysis, the hub genes of the candidate were enriched in 30 GO terms (Fig. 5A), such as protein processing in the endoplasmic reticulum, the unfolded protein response, and ER-associated degradation pathways were identified. These pathways are critical for maintaining cellular homeostasis and play a pivotal role in the pathogenesis of PAH by influencing endothelial function, smooth muscle cell proliferation, and inflammatory responses. Key genes within these clusters, such as SEC61B, EIF2S1, and DERL1, are involved in the translocation of newly synthesized proteins into the ER and the degradation of misfolded proteins. The dysfunction of these processes can lead to ER stress, a condition that contributes to vascular remodeling and pulmonary hypertension. For example, EIF2S1 is integral to the initiation of translation in response to ER stress and can influence cellular survival pathways, potentially impacting vascular stability and resistance to apoptosis in PAH. By connecting these functions to ER stress, we underscore potential therapeutic targets. Modulating the activity of pathways such as the unfolded protein response could offer new avenues for treatment aimed at alleviating ER stress and its downstream effects on pulmonary arterial pressure and vascular remodeling. Enrichment of candidate genes in four KEGG pathways was observed, including protein processing in the endoplasmic reticulum, amyotrophic lateral sclerosis, pathways of neurodegeneration in multiple diseases, and ubiquitin mediated proteolysis (Fig. 5B).

### Identification of hub genes

**Fig. 6:**
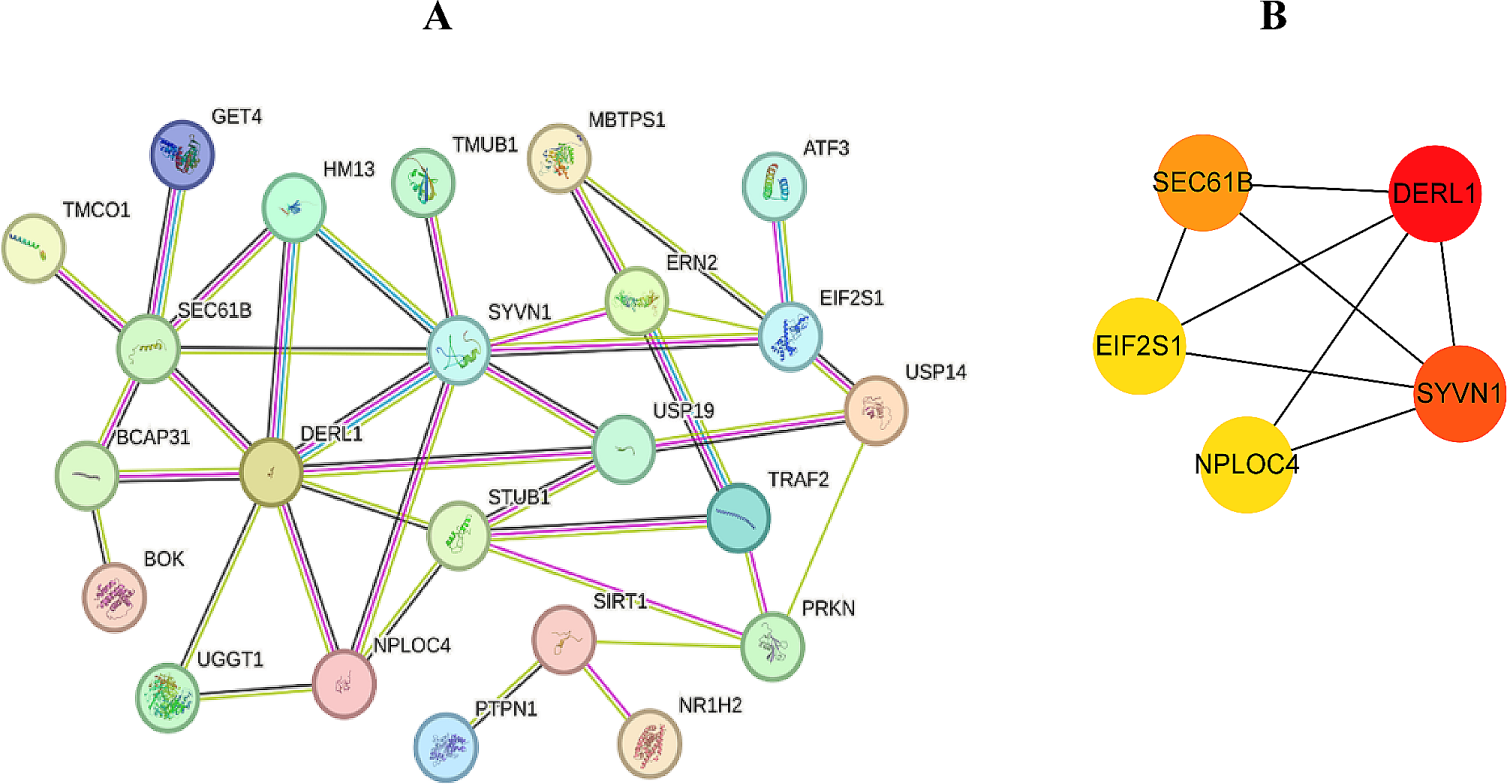
Protein-Protein Interaction (PPI) networks of hub genes related to pulmonary arterial hypertension (PAH). **A**: A detailed PPI network visualization highlighting the interactions among proteins encoded by the hub genes. Each node represents a protein, and the connecting lines represent the interactions with line colors indicating the types of evidence supporting the interaction. **B**: A simplified PPI network focusing on the five hub genes identified in the study. Nodes are color-coded based on their gene expression level with larger, darker nodes such as DERL1 and SYVN1 indicating a higher degree of differential expression. The thickness of the lines between the nodes reflects the strength of the data support for the interaction

**Fig. 7:**
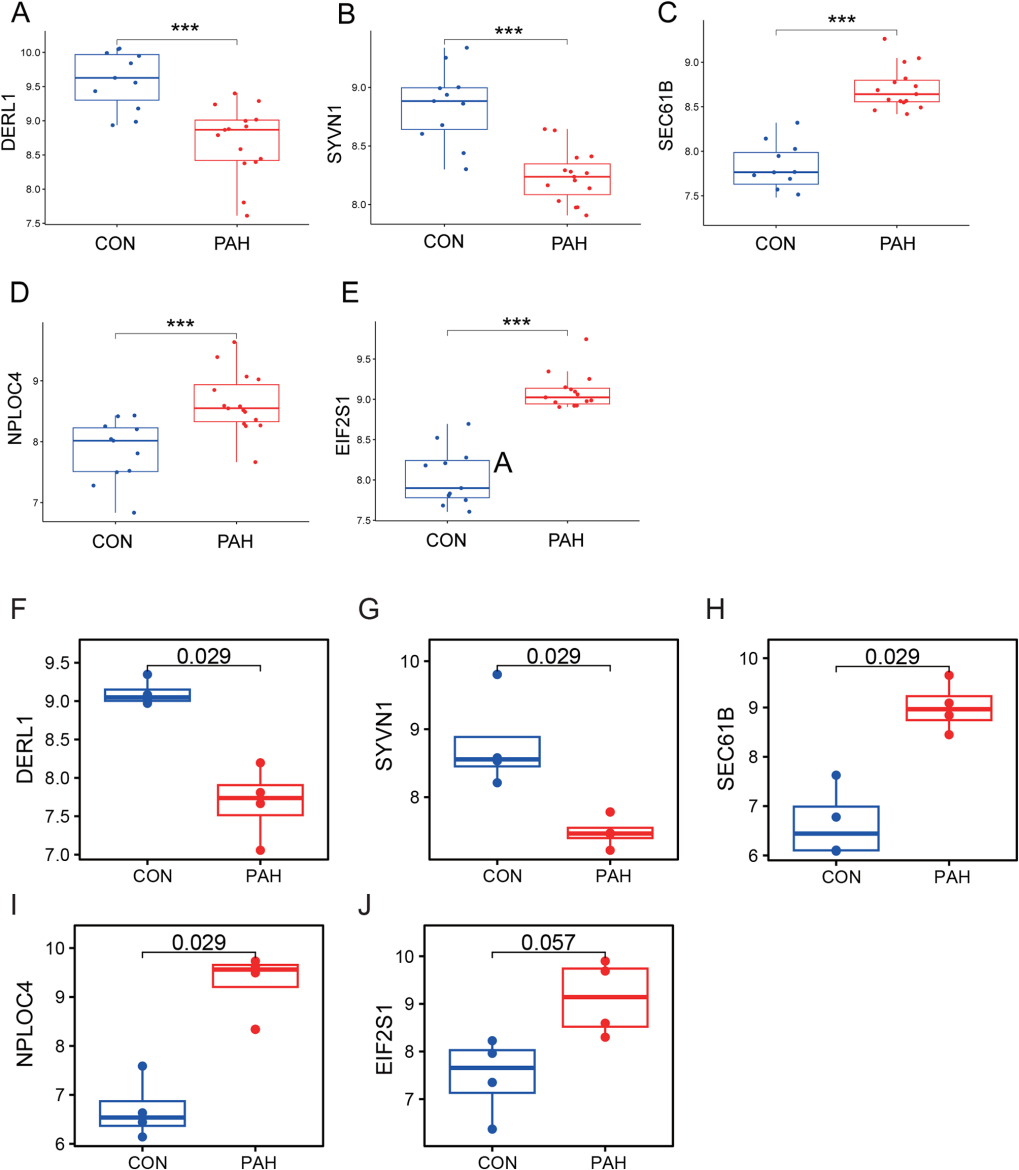
Expression levels of hub genes in control (CON) and pulmonary arterial hypertension (PAH) groups. **A-E**: Boxplots compare the expression levels of five key hub genes (A: DERL1, B: SYVN1, C: SEC61B, D: NPLOC4, E: EIF2S1) between control (CON) and PAH patient samples. Each panel represents one gene, In each plot, the y-axis indicates the expression level of the gene, and the x-axis categorizes the samples into control and PAH groups. The boxes encompass the interquartile range (IQR) of the data, with the median represented by the line within the box. Outliers are shown as individual points. The asterisks above the boxes denote the level of statistical significance, with ‘***’ indicating *p* < 0.001, illustrating a significant difference in expression between the groups. **F-J**: Boxplots compare the expression levels of five key hub genes (A: DERL1, B: SYVN1, C: SEC61B, D: NPLOC4, E: EIF2S1) between control (CON) and PAH patient samples from public data

**Fig. 8:**
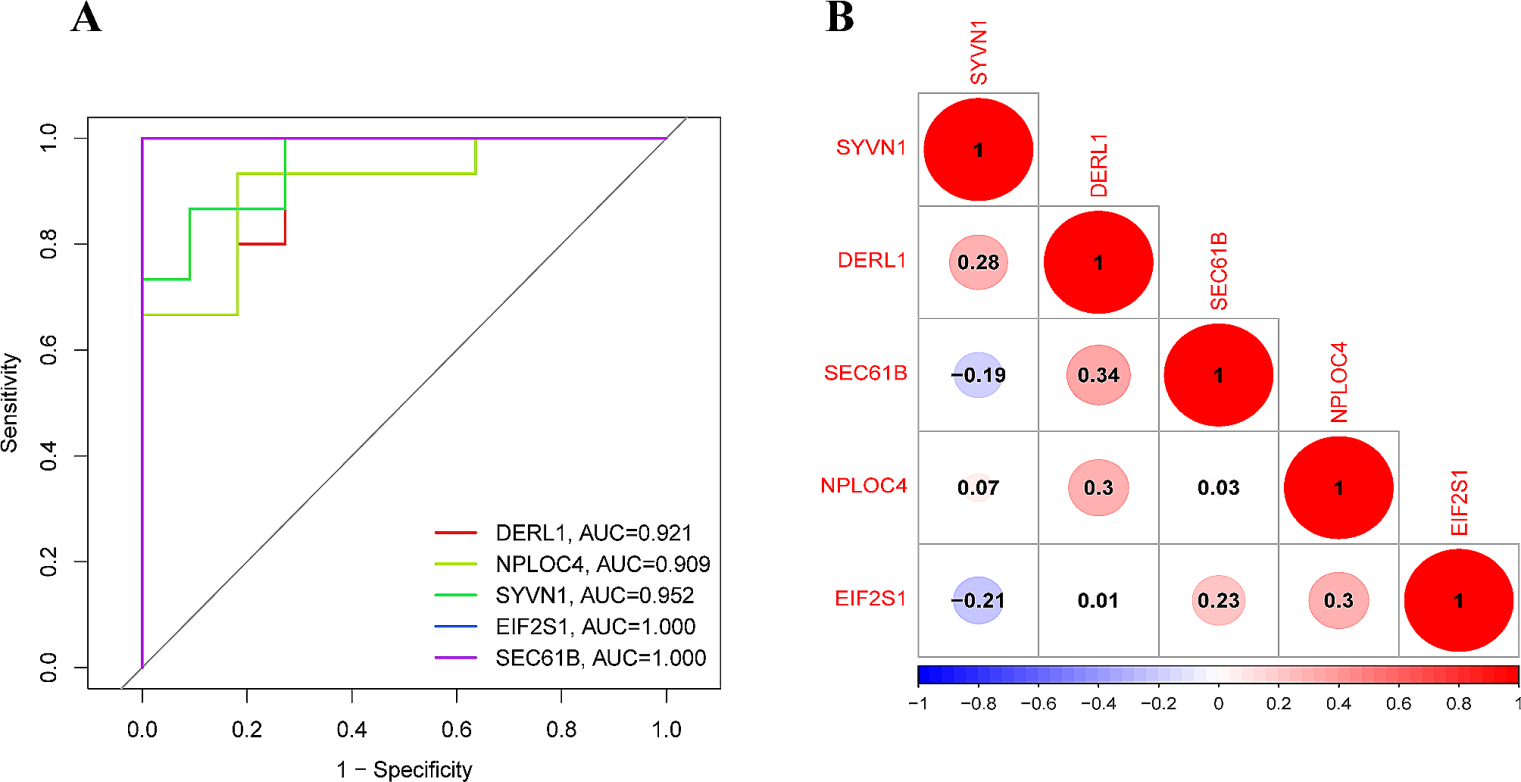
Evaluation of hub gene discriminative ability and correlation analysis in pulmonary arterial hypertension (PAH). **A**: Receiver Operating Characteristic (ROC) curves for each hub gene, assessing their performance in distinguishing between PAH and control groups. The Area Under the Curve (AUC) values are provided for each gene, indicating the accuracy of the gene as a potential biomarker for PAH. **B**: Correlation matrix showing the pairwise relationships between the hub genes. The size and color of the circles represent the strength and direction of the correlation, respectively, with red indicating a positive correlation, blue indicating a negative correlation, and color intensity reflecting the magnitude of the correlation coefficient

To identify the hub genes, a PPI network was constructed through the STRING online website (Fig. 6A), and 5 hub genes were obtained using the MCC algorithm in Cytoscape (Fig. 6B). In the GSE113439 dataset, there was differential expression observed in all 5 hub genes between the PAH and control groups. Controls exhibited higher expression levels of SYVN1 and DERL1 (Fig. 7). Based on ROC curve analysis, we identified five genes (EIF2S1, NPLOC4, SEC61B, SYVN1, and DERL1) that showed differential expression between PAH and control samples (Fig. 8A), for further analysis. Figure 8B indicated that SEC61B and DERL1 displayed the most robust positive correlation, whereas EIF2S1 and SYVN1 displayed the most robust negative correlation.

These genes form part of larger gene sets significantly associated with ER stress pathways that are pivotal in PAH development. For example, EIF2S1 and SEC61B are known to be involved in crucial processes such as the translocation of newly synthesized proteins into the ER and the degradation of misfolded proteins—key aspects of the unfolded protein response (UPR). Malfunctions in these processes can lead to exacerbated ER stress, contributing to the pathophysiological landscape of PAH by influencing endothelial dysfunction, smooth muscle cell proliferation, and inflammatory responses.

### Investigation of immune infiltration and its association with hub genes

**Fig. 9:**
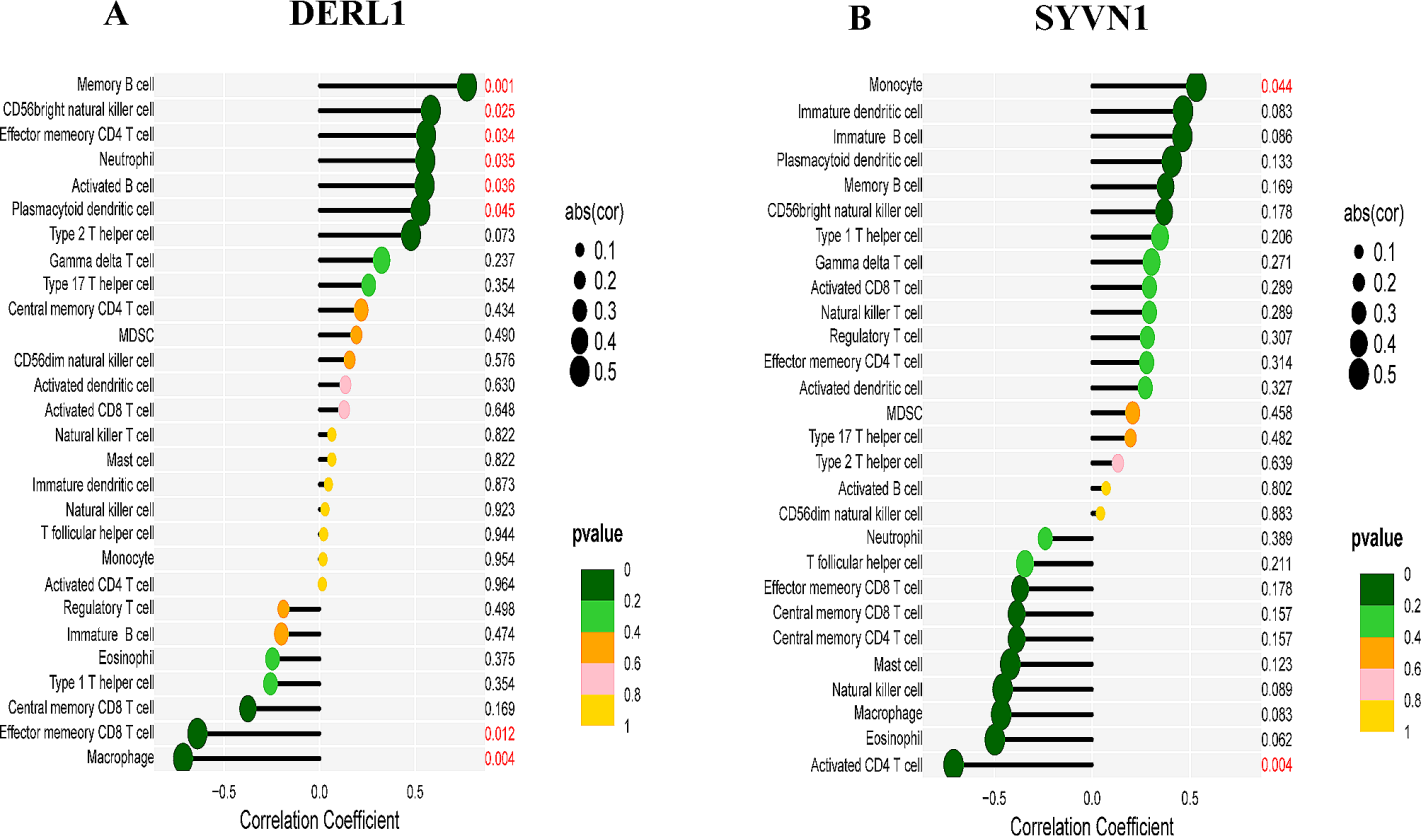
Correlations of immune cell infiltration scores with expression levels of hub genes. (**A**) DERL1; (**B**) SYVN1. The size of each dot is proportional to the absolute value of the correlation coefficient, while the color indicates the *p*-value, ranging from green (most significant) to red (least significant)

In order to examine the impact of hub gene expression on immune infiltration in PAH, we evaluated the relationship between the expression of central genes and the levels of infiltration by particular types of immune cells. The expression of DERL1 showed a strong correlation with eight different types of immune cells. In particular, the expression of DERL1 showed a positive correlation with memory B lymphocytes, CD56 bright NK cells, effector memory CD4 T lymphocytes, neutrophils, activated B lymphocytes, and plasmacytoid dendritic cells. In contrast, the expression of DERL1 showed a negative correlation with effector memory CD8 T cells and macrophages, as shown in Fig. 9A. Figure 9B showed a positive correlation between SYVN1 expression and monocytes, and a negative correlation with activated CD4 T cells. The expression of NPLOC4 showed a positive association solely with gamma delta T cells and did not demonstrate any significant correlation with other immune cell infiltrates. The expression of SEC61B showed a positive correlation with neutrophils and mast cells, but a negative correlation with regulatory T cells and monocytes. EIF2S1 expression showed no association with immune cell infiltration (see Supplementary Figure S2). These observations demonstrate multiple distinct immune microenvironments within tissue samples from PAH patients when compared to controls.

### Rat PAH model

**Fig. 10:**
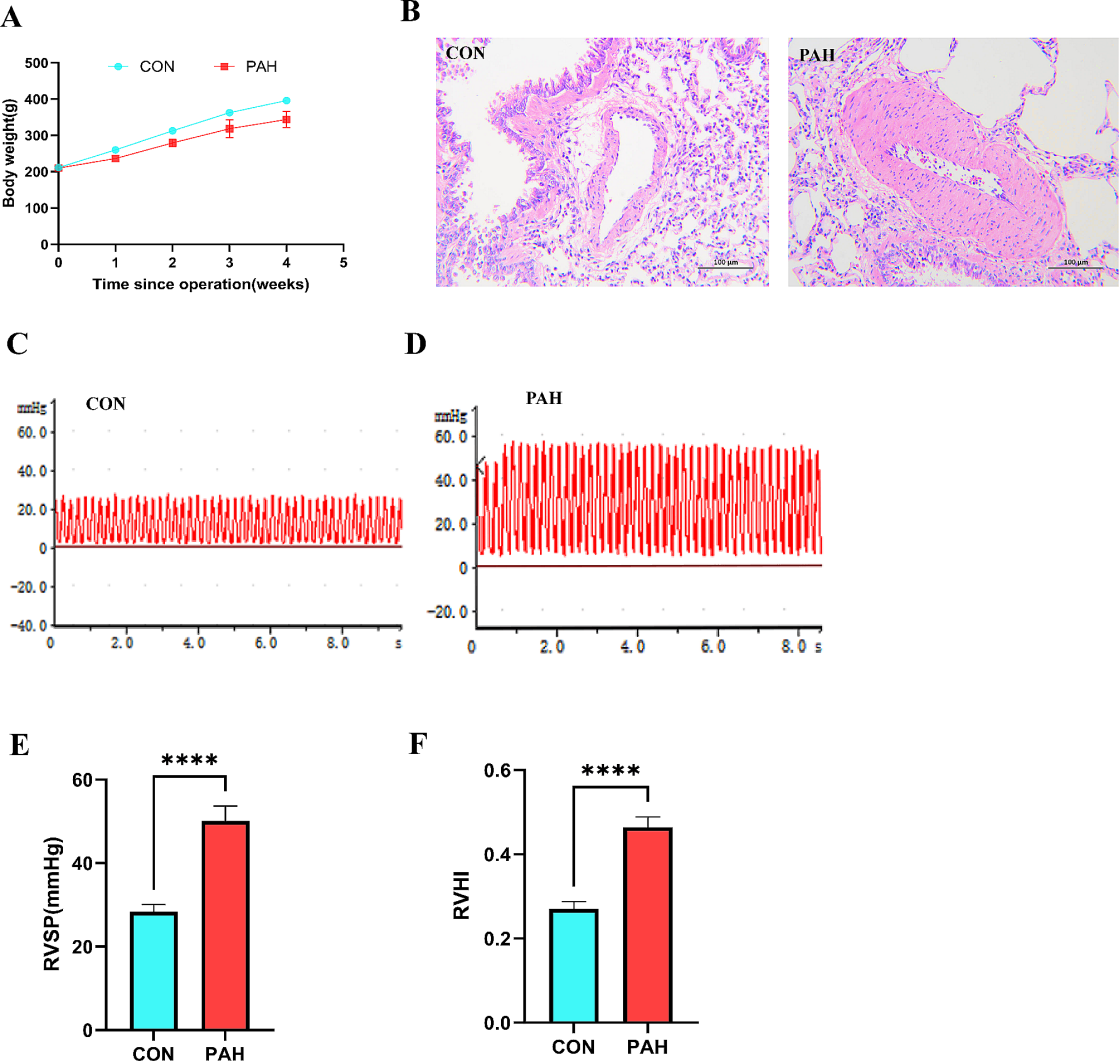
Longitudinal analysis of PAH progression and physiological assessment in a rat model. **A**: Body weight trajectory of control (CON) and PAH rats over a 5-week period post-operation, illustrating the weight gain or loss trends in both groups. **B**: Representative histological sections stained with hematoxylin and eosin (H&E) showing lung tissue from CON and PAH groups. The images provide a comparative view of the pulmonary architecture and potential pathological changes due to PAH. **C**: Electrocardiogram (ECG) trace from a control rat showing normal cardiac electrical activity. **D**: ECG trace from a PAH rat depicting alterations in cardiac electrical activity indicative of PAH-related heart stress or damage. **E**: Bar graph comparing the right ventricular systolic pressure (RVSP) between CON and PAH rats, a measure of the severity of pulmonary hypertension. **F**: Bar graph showing the right ventricular hypertrophy index (RVHI), a marker of heart muscle adaptation or strain in response to increased pulmonary arterial pressure, contrasting CON and PAH rats. **** in panels E and F indicates a statistically significant difference with a *p*-value < 0.0001 between the control and PAH groups

To confirm the successful establishment of the PAH model, we observed the rats in the PAH group for a duration of 4 weeks following a complete removal of the left lung. Throughout this time frame, it was noted that the rats in the PAH group exhibited a considerably reduced body weight compared to the control group (Fig. 10A). In the PAH group, right ventricular systolic pressure (RVSP) was markedly higher after 4 weeks than in the control group (Fig. 10C, D and E). The hemodynamic results were further confirmed by HE staining, which revealed notable vascular remodeling and thickening of the pulmonary artery wall in the PAH group in comparison to the control group (Fig. 9B). Moreover, the PAH group exhibited a significant increase in the right ventricular hypertrophy index (RVHI) compared to the control group (*p* < 0.05, Fig. 10F). The data collectively showed successful development of pulmonary hypertension and remodeling of the pulmonary blood vessels through complete removal of the left lung.

### Validation of hub genes

**Fig. 11:**
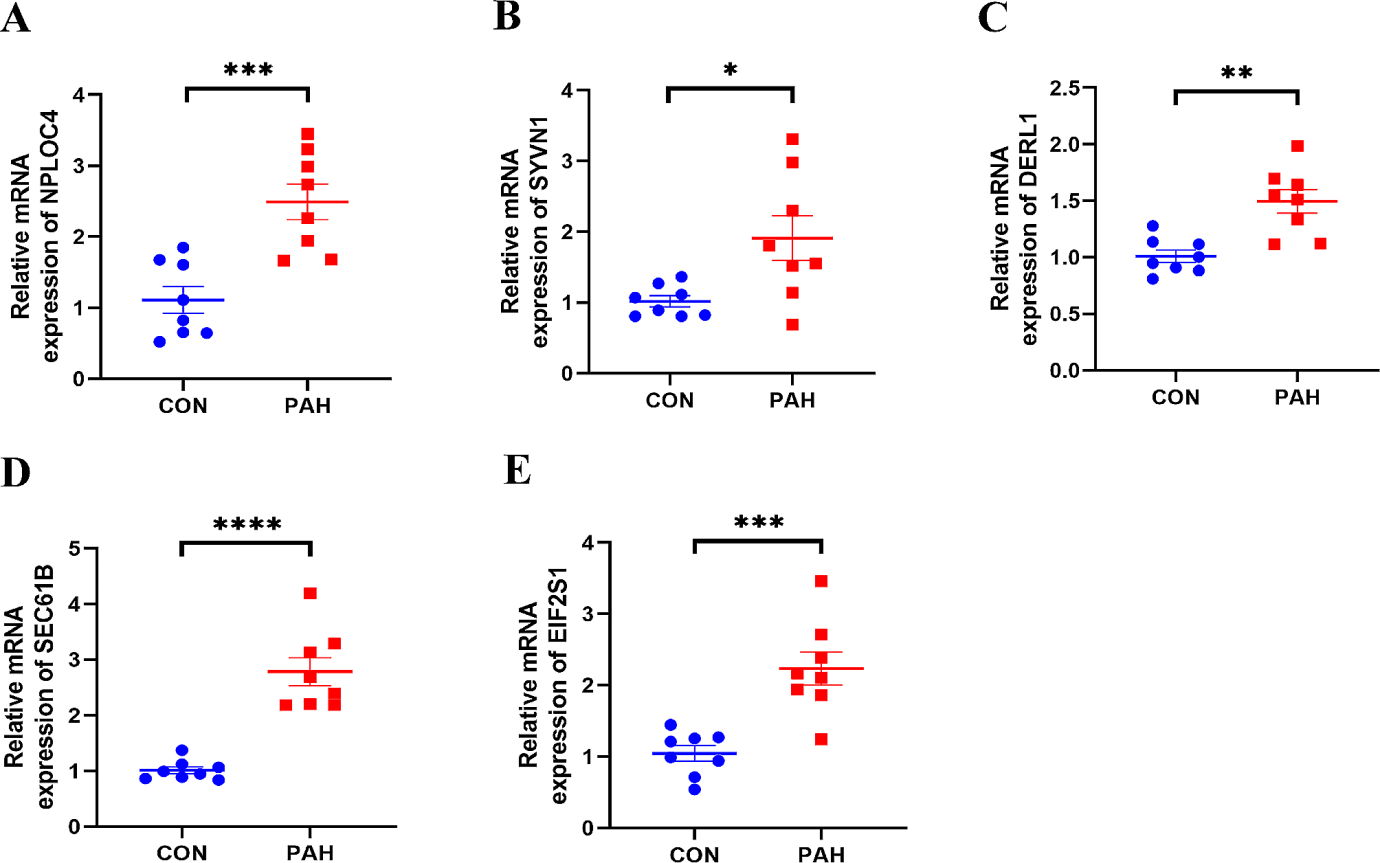
Expression levels of hub genes in control and pulmonary arterial hypertension (PAH) groups. Each panel (**A-E**) represents a scatter plot comparing the relative mRNA expression levels of a specific hub gene between control (CON) and PAH samples. The horizontal lines indicate the mean ± standard deviation of expression. **A**: Relative expression of NPLOC4. **B**: Relative expression of SYVN1. **C**: Relative expression of DERL1. **D**: Relative expression of SEC61B. **E**: Relative expression of EIF2S1. Statistical significance is denoted by asterisks: *p < 0.05, **p < 0.01, ***p < 0.001, ****p < 0.0001

After the onset of PAH, the expression levels of 5 hub genes were analyzed in lung tissues using qRT-PCR and experimentally validated. The expression pattern of SEC61B, NPLOC4, and EIF2S1 in PAH lung samples, as depicted in Fig. 11, aligned with the findings of bioinformatics analysis. However, DERL1 and SYVN1 exhibited contrasting trends in their expression (*p* < 0.05).

## Discussion

PAH is a serious disease affecting the pulmonary blood vessels, which has a major impact on the health and quality of life of patients. So far, the pathogenic mechanisms underlying PAH are still not fully elucidated and effective treatments remain lacking [20, 21]. Further investigation into the pathogenic basis of PAH and potential therapeutic targets is critically needed. Prior research has suggested that the occurrence of endoplasmic reticulum (ER) stress [22] and activation of immune cells, including macrophage polarization, infiltration of B-cells, and alterations in T cell subsets, play a role in the advancement of PAH [23]. Nevertheless, the precise impact of the combined regulation of ER stress and immune cell infiltration on the development and progression of PAH remains inadequately characterized. To uncover the pathogenesis and progression of PAH, this research employed bioinformatics analysis and experimental validation, examining the impact of ER stress and immune infiltration in combination.

To analyze ER stress, the study made use of MSigDB databases to identify genes associated with ER and utilized the WGCNA module to filter out 31 differentially expressed genes (DEGs) for further investigation. The analysis of functional enrichment showed that these differentially expressed genes (DEGs) in the ER were engaged in diverse biological processes associated with the ER, including reacting to ER stress, reacting to misfolded proteins, and binding to proteins at ubiquitin-like protein junctions. The pathway enrichment analysis indicated potential involvement of these genes in the development and advancement of PAH through pathways related to amyotrophic lateral sclerosis, neurodegeneration, protein processing in the endoplasmic reticulum, and protein hydrolysis mediated by ubiquitin. The genes comprised SEC61B, a fundamental element of the ER membrane protein translocation complex; NPLOC4, engaged in protein coding; EIF2S1, an initiator factor accountable for catalyzing protein synthesis; and DERL1 and SYVN1, coding genes implicated in ER-associated degradation. By utilizing the expression levels of these 5 hub genes, a diagnostic model was successfully able to differentiate patients from the controls with great accuracy and precision.

In earlier research, it was found that Let-7b-5p plays a role in the regulation of SERP1 and its associated protein SEC61B during the ER stress response, which affects the inflammation and apoptosis of lung cells in cases of acute pulmonary embolism [24]. Furthermore, the removal of SEC61B caused ER stress in both mammalian cells and Caenorhabditis elegans [25]. Moreover, it has been suggested that muscle atrophy could be alleviated by targeting the p97-NPLOC4 complex [26]. The identification of EIF2S1/eIF2α phosphorylation as a crucial mechanism helps regulate pathways for unfolded proteins and uphold intracellular homeostasis [27]. MDA-T68 cells showed significant upregulation of DERL1 in both thyroid cancer tissues and cell lines. Additionally, the overexpression of miR-575 resulted in the inhibition of DERL1 in these cells [28]. Hrd1, also referred to as Synviolin (SYVN1), was identified as one of the RING E3 ligases [29]. SYVN1 may control cell death caused by ER stress by facilitating the ubiquitination and breakdown of IRE1 [29]. To summarize, the involvement of these 5 hub genes in ER stress response suggests their potential contribution to various diseases. In order to experimentally validate these findings, we established a PAH rat model. The RT-qPCR findings demonstrated that the expression profile of SEC61B, NPLOC4, and EIF2S1 genes in the central genes aligned with the outcomes of bioinformatics analysis, justifying the need for additional exploration.

Immune infiltration is common in PAH and exacerbates the disease’s progression [18, 30, 31]. Increasing evidence indicates that signaling molecules associated with ER stress, such as ATF6, Eif2α, and CHOP, as well as inflammatory factors like IL-6, CCL-2, and MCP-1, are elevated in the pulmonary arteries of rats with PAH induced by wild larkin. Moreover, the application of clindamycin, an inducer of ER stress, leads to increased expression of inflammatory cytokines such as IL-6, IL-1β, and IL-2 in PASMCs. In contrast, the administration of Salubrinal, an inhibitor of ER stress, reduces the secretion of inflammatory molecules in PASMCs [32].

Furthermore, PAH is characterized by the chronic overexpression of Endothelin-1 (ET-1), a powerful peptide that constricts blood vessels, in both human and animal PAH models. When rat primary PASMCs are exposed to ET-1, it triggers the activation of unfolded protein response signaling pathway components like ATF6, Sxbp1, and eIF2α. This activation leads to the rapid buildup of ATF6 in the nucleus, which in turn stimulates the generation of pro-inflammatory factors in PASMCs, including interleukins and hyaluronic acid [33]. Additionally, our research revealed a notable correlation between hub genes associated with ER stress and the infiltration of immune cells in PAH. It is worth mentioning that the majority of the central genes showed a correlation with the infiltration of T cells and macrophages (refer to Supplementary Material S2). Consequently, our findings enhance the comprehension of the interplay between ER stress and immune cells in the context of PAH.

## Conclusion

Through bioinformatics analysis, we investigated the correlation between endoplasmic reticulum stress and the infiltration of immune cells in individuals diagnosed with PAH. By conducting experiments, we confirmed that three key genes (SEC61B, NPLOC4, and EIF2S1) have the potential to be molecular targets, providing valuable understanding of the mechanisms involved in PAH. However, there are constraints to our study. Initially, the main dataset was obtained from the GEO repository and we included only a restricted group of patients, emphasizing the necessity for validation in more extensive populations. Secondly, we confirmed the gene expression in rat models, but our study lacked corroborative clinical evidence. Furthermore, despite conducting comprehensive bioinformatics analysis, the study did not include experimental verification of how ER stress-related genes affect the immune microenvironment and the progression of PAH. Therefore, further exploration is needed to determine the specific functions of ER stress and immune infiltration in PAH, both in vivo and in vitro. This will be the primary focus of our future research efforts.

### Electronic supplementary material

Below is the link to the electronic supplementary material.

Supplementary Material 1: Supplementary Table S1. Primers sequence of hub ER-DEGs

Supplementary Material 2: Correlations of immune cell infiltration scores with expression levels of hub genes. EIF2S1; ERN2; HM13; NPLOC4; PRKN; SEC61B; STUB1; USP19; The size of each dot is proportional to the absolute value of the correlation coefficient, while the color indicates the *p*-value, ranging from green (most significant) to red (least significant)

## Data Availability

No datasets were generated or analysed during the current study.
