# Supplementary figures and images for: Identification of differentially expressed ER stress-related genes and their association with pulmonary arterial hypertension
Source: Respir Res. 2024 May 24;25:220. doi: 10.1186/s12931-024-02849-4 (PMC11127292; doi:10.1186/s12931-024-02849-4)

## EIF2S1

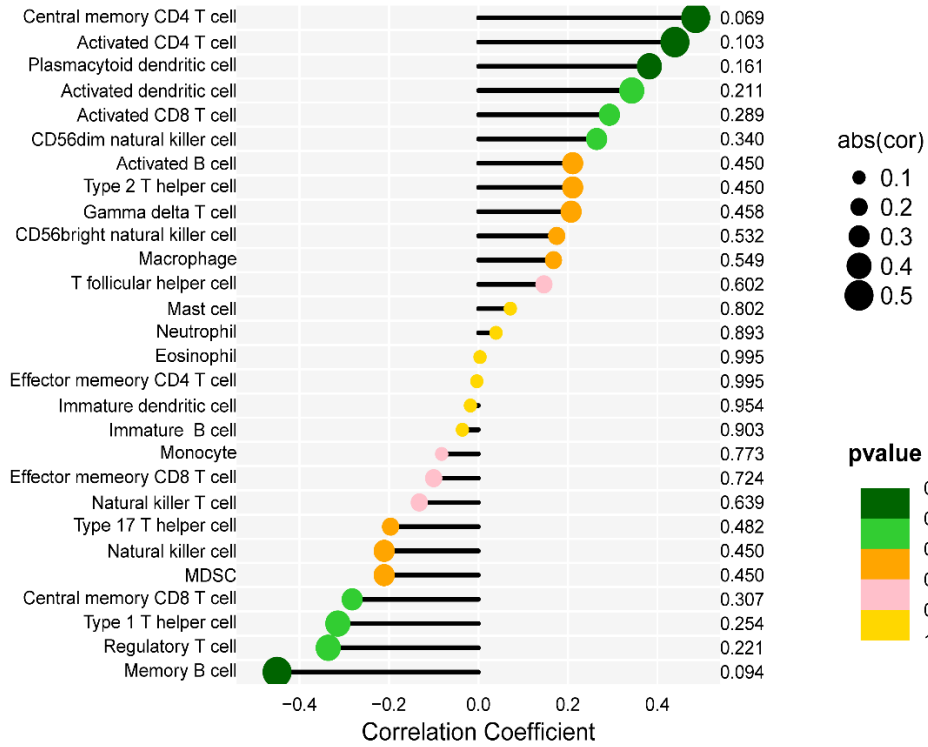

## ERN2

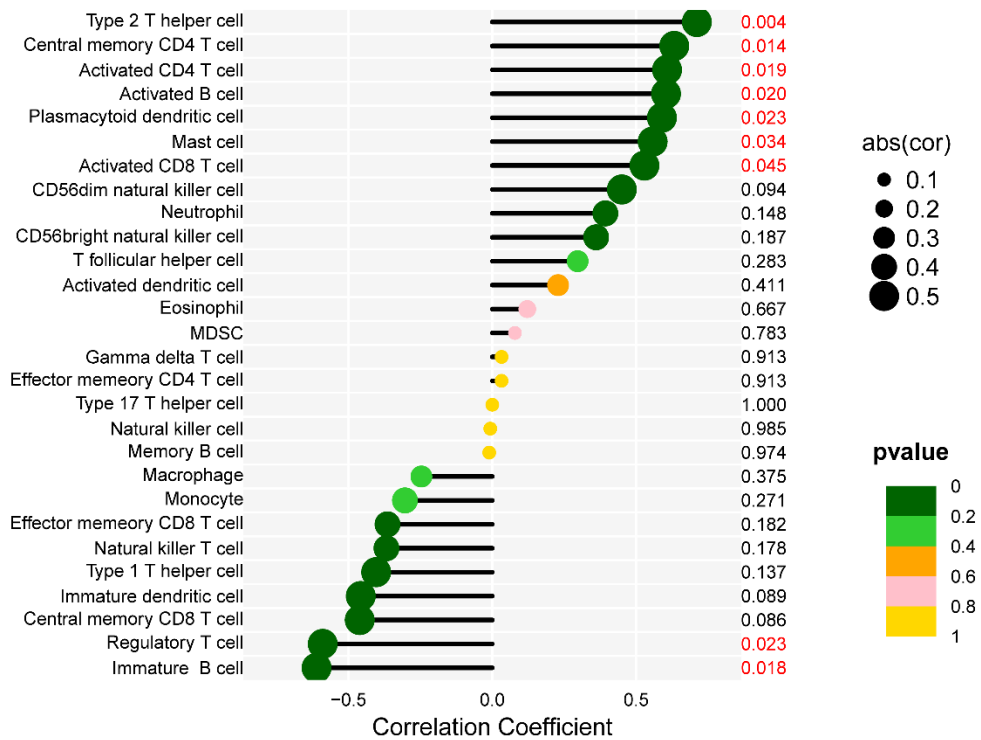

## HM13

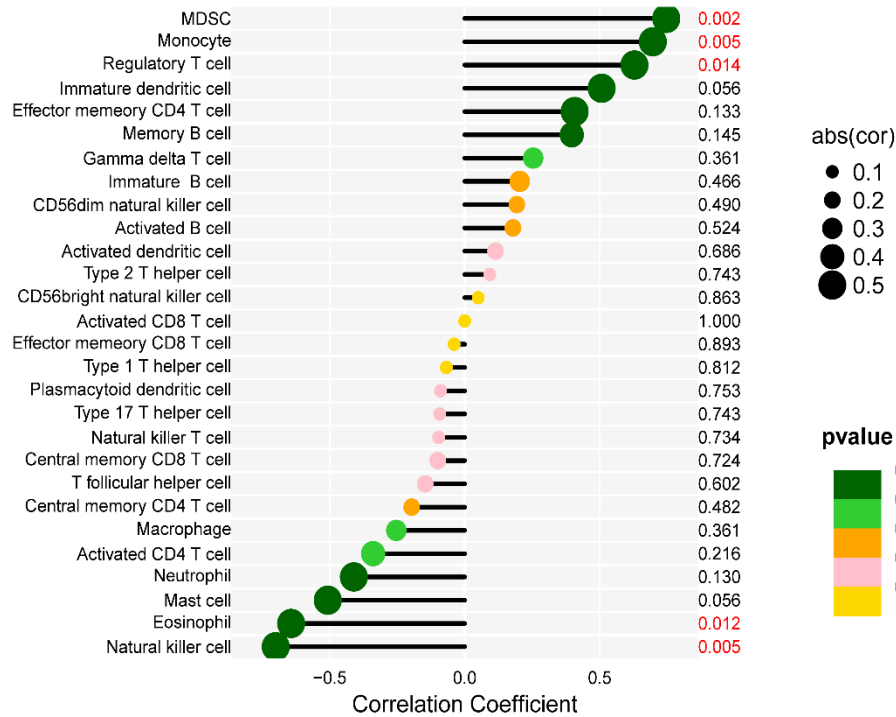

## NPLOC4

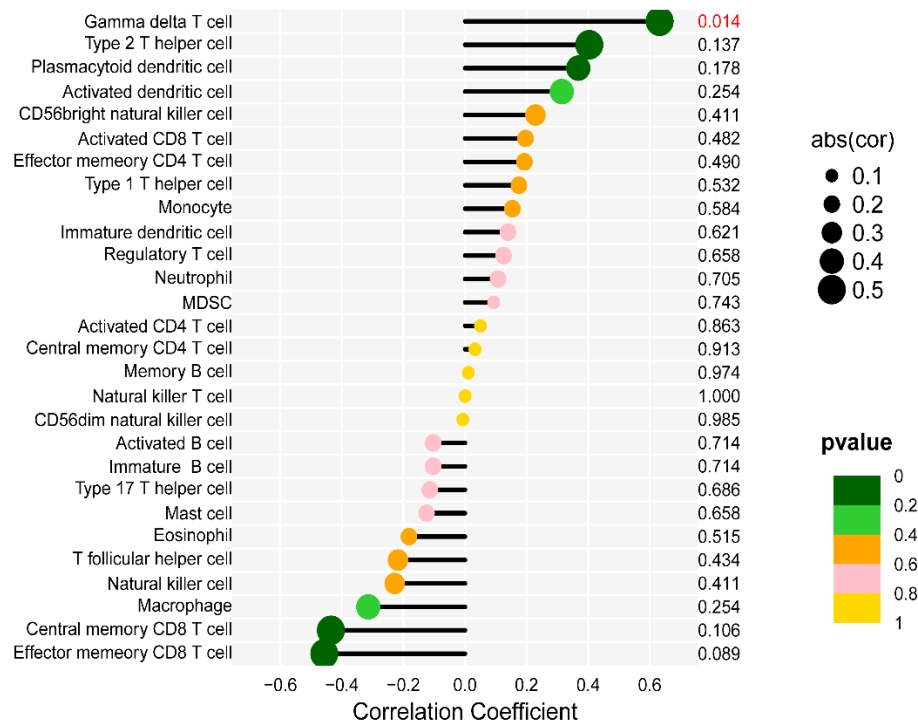

## PRKN

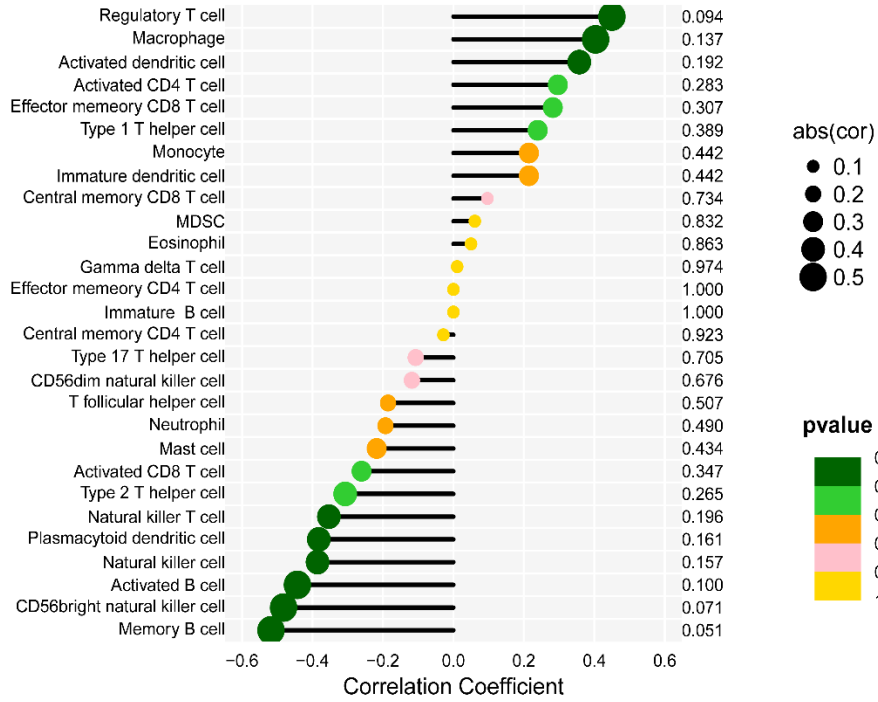

## SEC61B

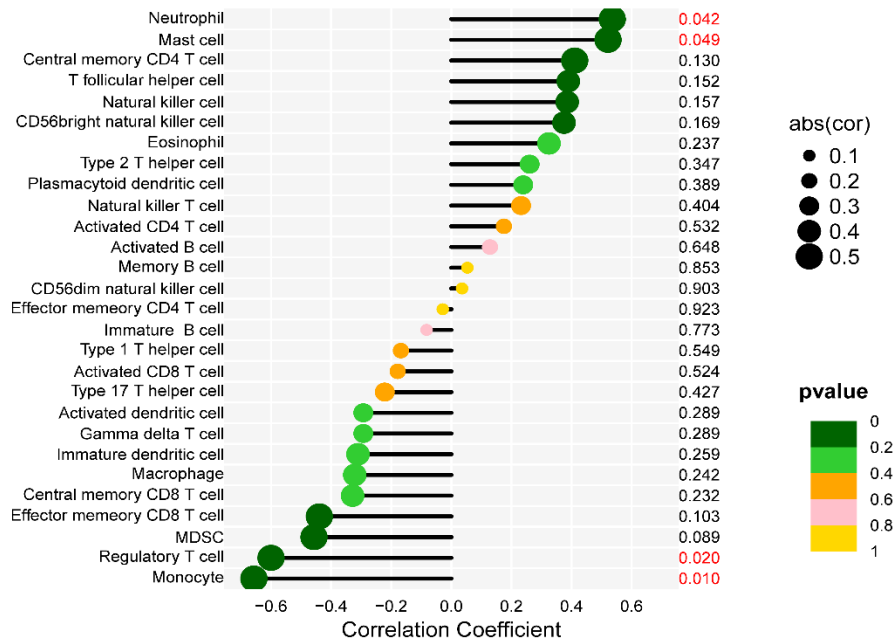

## STUB1

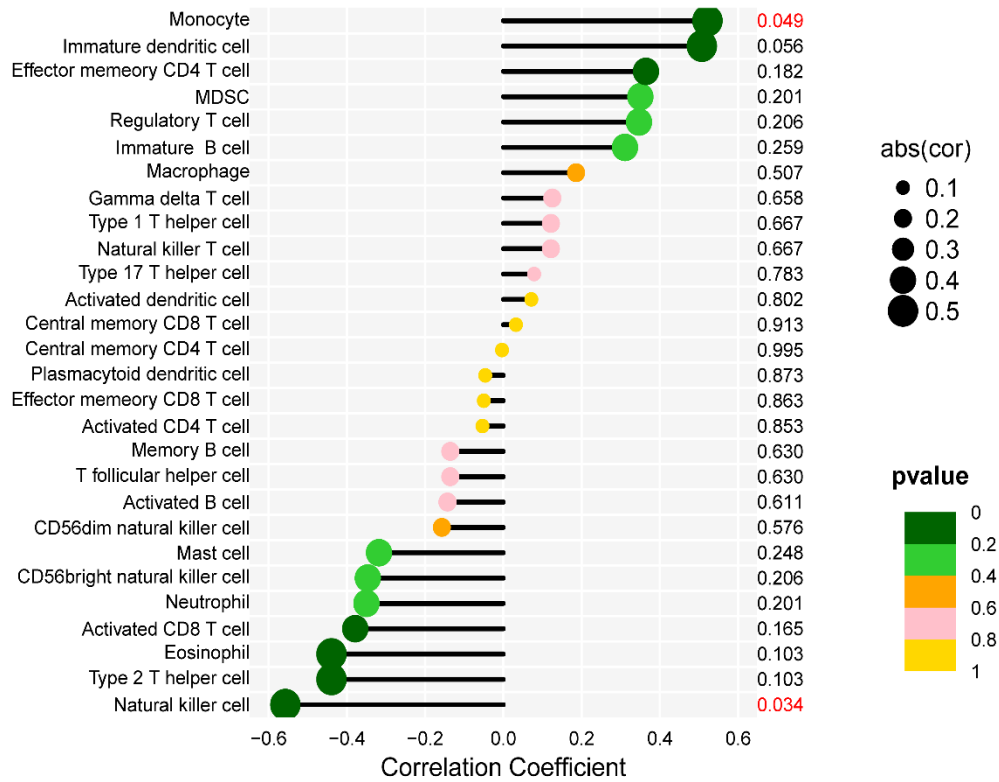

## USP19

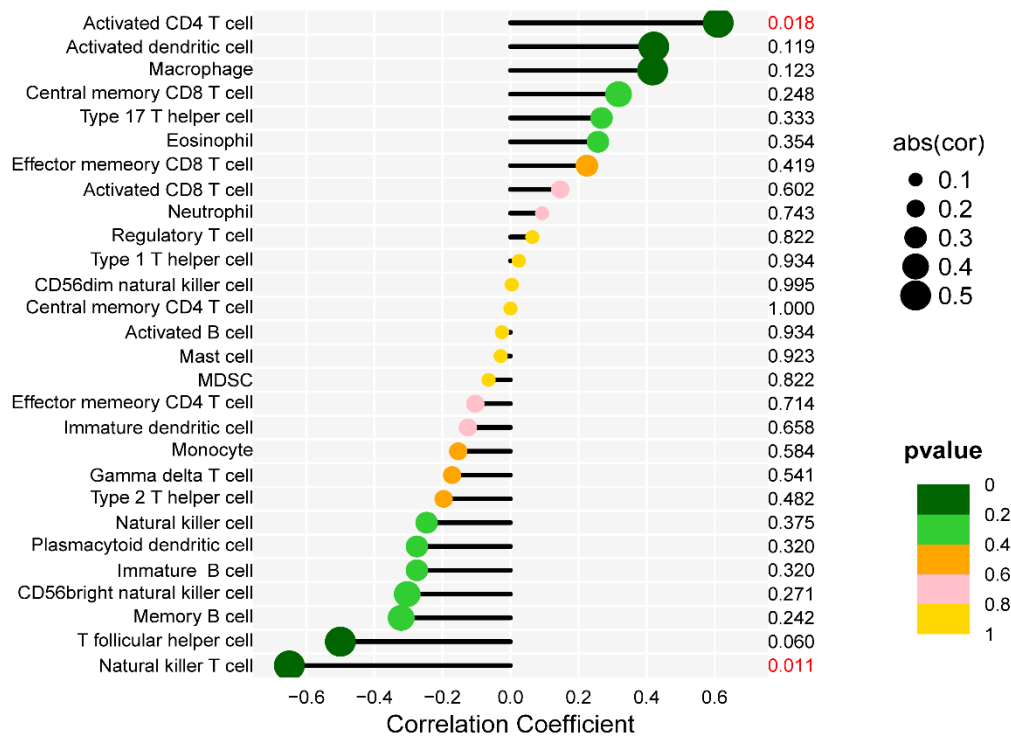

Supplement: Supplementary file 2 — Supplementary Material 2: Correlations of immune cell infiltration scores with expression levels of hub genes. EIF2S1; ERN2; HM13; NPLOC4; PRKN; SEC61B; STUB1; USP19; The size of each dot is proportional to the absolute value of the correlation coefficient, while the color indicates the p-value, ranging from green (most significant) to red (least significant) [file 12931_2024_2849_MOESM2_ESM.pdf]
